# Optimal Stimulus Shapes for Neuronal Excitation

**DOI:** 10.1371/journal.pcbi.1002089

**Published:** 2011-07-07

**Authors:** Daniel B. Forger, David Paydarfar, John R. Clay

**Affiliations:** 1 Department of Mathematics and Center for Computational Medicine and Bioinformatics, University of Michigan, Ann Arbor, Michigan, United States of America; 2 Marine Biological Laboratory, Woods Hole, Massachusetts, United States of America; 3 Wyss Institute for Biologically Inspired Engineering, Harvard University, Boston, Massachusetts, United States of America; 4 Departments of Neurology and Physiology, University of Massachusetts Medical School, Worcester, Massachusetts, United States of America; 5 National Institute of Neurological Disorders and Stroke, National Institutes of Health, Bethesda, Maryland, United States of America; University of Freiburg, Germany

## Abstract

An important problem in neuronal computation is to discern how features of stimuli control the timing of action potentials. One aspect of this problem is to determine how an action potential, or spike, can be elicited with the least energy cost, e.g., a minimal amount of applied current. Here we show in the Hodgkin & Huxley model of the action potential and in experiments on squid giant axons that: 1) spike generation in a neuron can be highly discriminatory for stimulus shape and 2) the optimal stimulus shape is dependent upon inputs to the neuron. We show how polarity and time course of post-synaptic currents determine which of these optimal stimulus shapes best excites the neuron. These results are obtained mathematically using the calculus of variations and experimentally using a stochastic search methodology. Our findings reveal a surprising complexity of computation at the single cell level that may be relevant for understanding optimization of signaling in neurons and neuronal networks.

## Author Summary

Computational neuroscience seeks to understand the mechanisms by which signals excite a neuron or a neuronal network. An important consideration in these studies is optimality, i.e., what signal most effectively causes excitation. Optimization of neuronal signaling is important for networks that need to minimize energy costs, for sensory neurons to selectively respond to specific stimulus features, and for therapeutic deep brain stimulators to maximize battery life. Here we show in a classic mathematical model of the action potential and in experiments on a single cell preparation that: 1) a single neuron can be highly discriminatory for the shape of low amplitude stimuli that elicit an action potential and 2) the shape of the optimal stimulus depends upon the overall state of inputs to the neuron. Our findings reveal a surprising complexity of computation at the single cell level that may be important for understanding physiological function of the nervous system.

## Introduction

A central question in neuronal computation is to determine the features of neural stimuli that cause action potentials [1], [2]. One aspect of this problem is a study of how an action potential, or spike, can be elicited by a signal with the least energy cost, e.g., a minimal amount of applied current [3], [4]. This problem is relevant to a number of questions in neuroscience, e.g., what mechanisms enable sensory neurons to optimally discriminate between different percepts [5], [6], and what are the optimal shapes of exogenous current stimulations that cause excitation in a neuronal network for therapeutic purpose [7]–[9]. Here we investigate stimulus optimization in a well-studied neuronal preparation using computational and experimental methods.

One method for determining optimal signals is the calculus of variations [10]. The rationale of this approach is that if a particular signal is optimal, small changes in signal shape cannot lead to a more effective signal for eliciting a desired response. This requirement allows a determination of relative optimum shapes. Another approach for finding optimal stimuli uses a stochastic search methodology [11]. In this method an array of stochastically determined stimulus shapes is considered, including those that displace the membrane from rest to firing. When the overall intensity of the stimulus array is reduced to a level at which action potentials rarely occur, then such rarely supra-threshold stimuli are candidate optimal shapes for eliciting an action potential. Comparison of these methods has yielded similar optimal stimulus shapes in models of biological oscillators [11].

**Figure 1:**
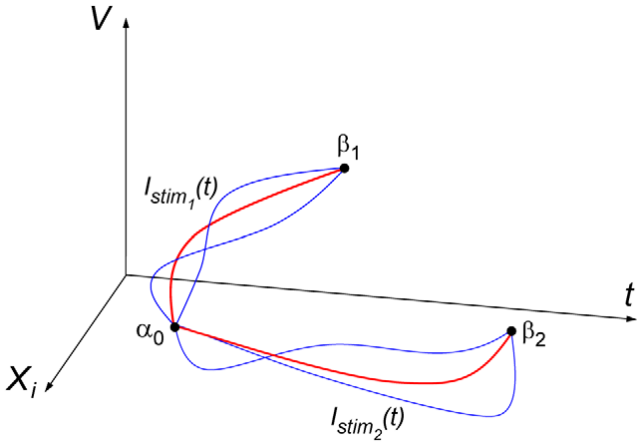
Explanatory diagram of the effect of current stimulations on trajectories of neuronal variables *V*, membrane potential, and the neuron's i^th^ state variable, *X*_i_, such as one of the ion channel gating parameters. Stimulation trajectories *I*stim_1_ induce a state change from rest α_0_ to threshold β_1_ by means of depolarization, and stimulation curves *I*stim_2_ induce a state change to β_2_ by means of post-inhibitory rebound. Red trajectories illustrate the optimal paths for which total current is minimized; blue trajectories are neighboring paths of suboptimal stimulation.

An important step in addressing these questions is the development of a theory of optimality in single neurons. This theory should account for the complex, multi-scale and nonlinear behavior of a neuron. For example, several mechanisms are known to generate an action potential including membrane depolarization and post-inhibitory rebound excitation [2], as illustrated in Figure 1. A family of neighboring trajectories exists for each mechanism that takes the neuron from rest to an action potential. We seek for each mechanism the optimum trajectory that triggers an action potential with the least energy cost, for example the total current delivered.

The signals a neuron receives are combinations of post-synaptic currents (PSCs), which can be either excitatory or inhibitory. The duration of PSCs can vary considerably depending on cell type [12]. Moreover, the timing, number, and amplitude of PSCs also vary significantly. Consequently, PSCs can, in principle, generate a wide range of signals in the post-synaptic cell, although the properties of the post-synaptic cell limit the output that the cell can actually produce. A theory of neuronal optimality should account for these physiological constraints.

In the present study we investigate stimulus optimization principles using one of the best characterized experimental preparations - the squid giant axon - and its mathematical representation, the Hodgkin & Huxley model and a recent modification of the model [13], [14]. A major finding is that the excitatory properties of this preparation are, as suggested above, exquisitely sensitive to stimulus shape. Moreover, the neuron uses different mechanisms for generating an action potential depending on the physiological context in which it finds itself thereby requiring context dependent optimal shapes. These results on stimulus optimization in single neurons may be important for considering optimization within and across neural circuits throughout the nervous system.

## Results

### Stimulus optimization in the Hodgkin & Huxley model

**Figure 2:**
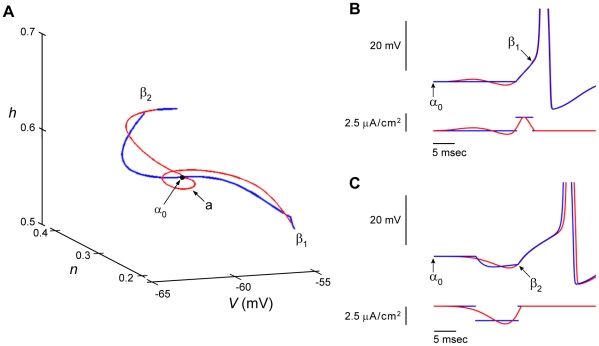
Two distinct mechanisms of neuronal excitation in the Hodgkin & Huxley model. **A**) Three dimensional phase representation of state variables (*h*, sodium channel inactivation; *n*, potassium channel activation; *V*, membrane potential). Depolarizing stimulation currents induce a state change from rest α_0_ to threshold β_1_ and post-inhibitory rebound stimulation induces excitation via state change from rest α_0_ to threshold β_2_. Blue trajectories are paths corresponding to rectangular current pulses; red trajectories are the optimized paths computed from the model with the calculus of variations. Note the small loop (**a**) of the optimized depolarizing trajectory. **B**) and **C**) illustrate the two mechanisms of excitation using the same depolarizing and post-inhibitory rebound stimulations shown in **A**). Membrane potential changes elicited by rectangular pulses are shown in blue; changes elicited by the stimuli calculated from the calculus of variations are shown in red. Further details given in the text.

The Hodgkin & Huxley model [13] consists of four state variables, *V*, *m*, *h*, and *n*, where *V* is membrane potential, *m* and *h* are associated with the sodium ion current, *I_Na_*, and *n* is associated with the potassium ion current, *I_K_* (Methods). The model provides an excellent description of the action potential response of squid giant axons to suprathreshold depolarizing current pulses having brief duration. It is less successful for longer duration pulses. In particular, it predicts repetitive firing for these conditions over a large range of pulse amplitudes. The axon itself fires once and only once regardless of pulse amplitude or duration [14]. This discrepancy between theory and experiment is accounted for by changing a single parameter in the equation for *n*, the *I_K_* gating variable [14]. Both versions of the model provide comparable descriptions of the response of the axon to brief duration pulses, which is the focus of this work, i.e., the optimal stimulus for eliciting a single spike rather than a train of spikes. Consequently we begin our analysis with the original version of the model and compare those results with results obtained from the revised version. All simulations were carried out with the full model (either version) including results obtained using calculus of variations (Methods). We elicited an action potential in the usual way, i.e., with a rectangular depolarizing current pulse *I_stim_*(*t*) having slightly suprathreshold amplitude (Figure 2B, blue tracings). The pulse takes the model from the rest state α_0_ to threshold β_1_ along the trajectory in *V*, *n*, and *h* space illustrated in Figure 2A (blue tracing). We used the calculus of variations to find a neighboring *I_stim_*(*t*) trajectory that also takes the model from α_0_ to β_1_ with a minimum amount of applied root mean square (RMS) current (red tracing in Figure 2A). The *V* vs *t* result obtained is overlaid on the rectangular pulse result in Figure 2B. The RMS current of the calculus of variations stimulus over its 20 msec duration is approximately 40% less than that of the 4 msec duration rectangular pulse. We note that the stimulus obtained from the calculus of variations contains an oscillatory component, seen as a loop around α_0_ in Figure 2A (arrow) coinciding with the oscillations in stimulus current and membrane potential shown in Figure 2B. As noted above, action potentials are also elicited following a hyperpolarizing current pulse - anode break excitation, a result referred to as post-inhibitory rebound (PIR). These conditions partially remove the resting level of *I_Na_* inactivation by increasing the *h* state variable from its resting level. The effect of a hyperpolarizing pulse on the *h* variable is the mechanism underlying PIR in the Hodgkin & Huxley model. We adjusted the current amplitude of a 10 msec hyperpolarizing pulse until threshold was achieved, state β_2_ in Figure 2A. Note that the membrane potential of β_2_ at the end of the hyperpolarizing pulse is below the resting level (Figure 2C). Referring to this point as a threshold for spike initiation may seem counterintuitive but is consistent with the behavior of both the Hodgkin & Huxley model and squid giant axons. A hyperpolarizing pulse of insufficient amplitude or duration will fail to elicit an action potential following the pulse. Increasing both, or either, pulse parameter will generate a spike. We fixed the pulse duration at 10 msec and increased its amplitude until a spike was elicited. The *V*, *n*, and *h* trajectory of this result connecting α_0_ and β_2_ is illustrated in Figure 2A (blue tracing). The calculus of variations was used to identify a nearby trajectory (red tracing in Figure 2A connecting α_0_ and β_2_) that minimized the amount of current required for the anode break result. The *V* vs *t* tracings for both results are overlaid in Figure 2C. In this case the RMS current throughout its 20 msec duration is ∼22% less than that of the 10 msec duration rectangular pulse. Note that the timing of the action potential elicited by the pulse in Figure 2C does not exactly match that of the spike elicited by the calculus of variations signal even though both waveforms do closely overlap for some time following each respective stimulus. This result is attributable to the non-linear character of the Hodgkin & Huxley model. (The blue and red voltage waveforms more nearly superimpose in Figure 2B.) For both sets of results in Figure 2 the calculus of variation trajectory was optimal relative to the trajectory corresponding to a spike elicited by an excitatory or inhibitory rectangular pulse.

In the above analysis the only restrictions placed on the current *I_stim_*(*t*) using the calculus of variations is that it takes the Hodgkin & Huxley model from point α_0_ to β_1_ (or β_2_) in 20 msec with minimal RMS current. This approach is relevant for exogenous stimulation of a neuron that occurs, for example, during deep brain stimulation [7]–[9] in which *I_stim_*(*t*) is unconstrained by the intrinsic properties of the membrane. Neuronal PSCs generated endogenously are constrained by the ionic mechanisms of excitability expressed generically for a synapse by the relationship *I_stim_*(*t*) = *g_syn_*(*t*) (*V*(*t*)-*E_syn_*). We used calculus of variations to find the optimal pathway to a spike - optimal *g_syn_*(*t*) - with either excitatory or inhibitory PSCs. These results (supporting material: Text S1, Figures S1 and S2) are not substantially different from the results in Figure 2 at least when *E_syn_* is far from the membrane potential *V*(*t*). We note that more complex stimuli can be seen when *V*(*t*) is close to *E_syn_*. In the remainder of this study we optimized exogenous *I_stim_*(*t*) since our experimental protocol explicitly tests for candidate optimal stimuli applied to the membrane. The calculations below (supporting material) suggest that the approach we are using is relevant to at least a range of endogenous synaptic currents.

### Optimization using noisy perturbations

**Figure 3:**
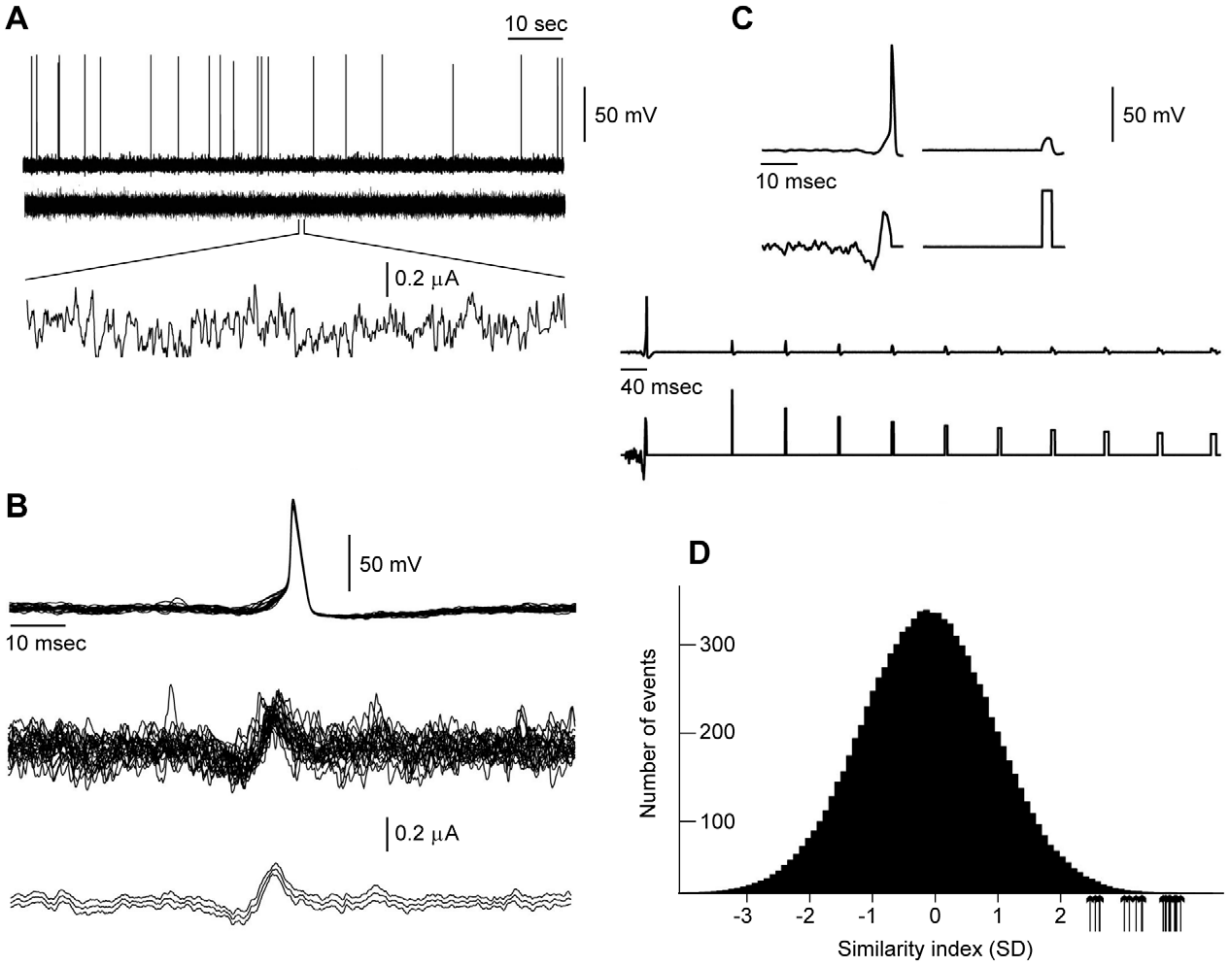
An example of a search for stimulus shapes that optimally excite the squid giant axon preparation using the stochastic approach described in the text. These results are all from a single axon. Similar results were observed in all other axons. **A**) The axon was stimulated with a stochastically varying time series consisting of modeled excitatory and inhibitory post-synaptic currents (PSCs), as described in the text. Shown is a stimulation trial of 100 seconds consisting of balanced excitatory and inhibitory PSCs (zero mean current) having a mean Poisson rate of 1 msec^−1^. Each PSC had a decay constant of 1 msec. The stimulation amplitude (RMS) was set to a level that produced rare action potentials (<1 Hz). **B**) All twenty-one action potentials from the trial were superimposed by aligning their peak voltages, i.e., the maximum overshoot potential. The corresponding input currents were similarly aligned (middle tracing). The bottom panel illustrates the mean (±2 SEM) of the stimulus currents. This spike-triggered average is the candidate optimal stimulus shape. **C**) The candidate optimal stimulus from **B** elicited an action potential when it was administered to the axon (first tracing with the stimulus shown below the action potential response). The stimulus is the same as in the bottom panel of **B**. A rectangular depolarizing current pulse having the same RMS current as the optimal stimulus failed to elicit an action potential (top tracing to the right of the action potential). Rectangular pulses having durations ranging from 1 to 10 msec with the same RMS current as the optimal stimulus failed to elicit action potentials (bottom two tracings). **D**) Histogram generated by convolving the optimal stimulus in **B** with the raw stimulus in **A**, as described in the text. This convolution measures how similar the optimal is to any 20 msec portion of the input signal (**A**, tracings below the action potentials elicited by the input). We refer to this as similarity index. This parameter is measured in units of standard deviations (SD) from the mean. Action potentials were seen only when this measure was >2 SD above the mean as indicated by the arrows showing that stimuli that elicited action potentials were highly similar in shape to the optimal.

As noted above, stochastic perturbations can also be used to determine stimulus optimization without requiring a mathematical description of the underlying dynamics [11]. We implemented the stochastic approach experimentally using squid giant axons. We used noise that consisted of excitatory and inhibitory model PSCs having rise and decay time constants based on experimental data [12]. In any given experimental run PSC shapes were kept the same. The times at which PSCs were added to the input signal were determined using a random number generator. Figure 3 illustrates an example of our experimental protocol along with results obtained from a single axon. A 100 sec stimulus was applied that consisted of PSCs having a one millisecond decay constant where excitatory and inhibitory PSCs were generated with equal probability. The details of the stimulus are illustrated in the bottom trace of Figure 3A which is a one second portion of the signal shown on an expanded time scale. The intensity of stimulation was adjusted to so that spikes were elicited infrequently (0.05–1 Hz) as required by the stochastic search methodology [11]. We analyzed the portions of the run during which spikes were elicited to determine the specific attributes of the stimulus that preceded the action potentials. All spikes were aligned at the time of their peak voltage (Figure 3B, top panel). The underlying stimulus currents were similarly aligned so that a spike-triggered average of the stimulus could be obtained (Figure 3B, middle panel). The average values of the current (±2 SEM) are shown in the bottom panel of Figure 3B. Based on previous work [11], we hypothesized that the average stimulus prior to the spike is an optimal stimulus shape, i.e., this signal should elicit an action potential with minimal current. To test this hypothesis, we applied this stimulus to the same axon from which the results in Figure 3A were obtained and found that it did, in fact, elicit an action potential (Figure 3C). Note that the candidate optimal stimulus in the bottom panel of Figure 3B is shown in Figure 3C on a different time scale below the action potential elicited by the stimulus. A rectangular depolarizing current pulse having the same RMS current amplitude as the optimal stimulus failed to elicit an action potential (Figure 3C). The comparison of the effects of the experimentally determined optimal stimulus with rectangular pulses is further illustrated in the bottom tracings of Figure 3C shown on a compressed time scale relative to the results in the top panels of Figure 3C. Rectangular pulses having the same RMS current amplitude as the optimal stimulus and with durations ranging between 1 and 10 msec were applied to the axon. None of the pulses elicited a spike.

Next we asked how close any 20 msec portion of the input signal (Figure 3A) was to the shape of the optimal stimulus (similarity index). To do this we considered the 20 msec of signal prior to each time point and convolved these signals with our time-reversed candidate optimal signal. The results are given by the histogram in Figure 3D which is very close to a Gaussian distribution. We then considered the 20 msec signals that preceded each action potential. Every one of these signals that elicited an action potential (shown by arrows in Figure 3D) were greater than 2 standard deviations from the mean in this histogram, indicating a high correlation with the optimal stimulus. This result also indicates that the optimal stimulus has strong predictive value in determining when the axon will fire.

**Figure 4:**
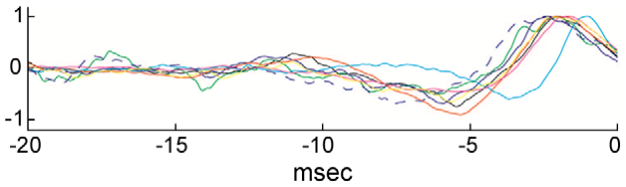
Optimal stimulus for eliciting a spike using the procedure described in **Figure 3** for all seven preparations for which this experiment was carried out. Each signal was normalized to its maximum value that occurred within a few msec before a spike.

The experimental protocol and analysis illustrated in Figure 3 was carried out on a total of seven axon preparations. In all seven we confirmed the results shown in Figure 3B–D. The optimal noise stimuli obtained from each experiment (including the result in Figure 3C) are shown superimposed in Figure 4.

### Comparison of the noise and calculus of variations methods

A visual comparison of the noise-derived optimal signal in Figure 3C with the calculus of variation waveforms in Figure 2B & C reveals important differences in stimulus shape. The result in Figure 2B has a marked depolarization phase early in the signal that is not clearly apparent in the experimental results obtained with the stochastic approach (Figures 3C & 4). The experimental results have two clear phases: a marked hyperpolarization followed by depolarizing phase just prior to spike initiation. Not surprisingly, therefore, neither of the waveforms in Figure 2 elicited a spike from the axon preparation described in Figure 3 when the RMS amplitude was adjusted to match the RMS level of the noise stimulus (results not shown). In other words, the noise-derived optimal shape was superior to the shape derived from the calculus of variations. We hypothesized that these differences between the experimental and theoretical results might be attributable to our observation that rectangular pulses do not optimally elicit spikes (Figure 3C). The initial conditions used for the waveforms in Figure 2B & C were the set of values for *V*, *m*, *h*, and *n* corresponding to rest - the starting point for the calculus of variations - and the set of values for *V*, *m*, *h*, and *n* corresponding to the end of rectangular pulses - the end point for the calculations. Since rectangular pulses do not themselves optimally elicit spikes, the observation that the values of the Hodgkin & Huxley model obtained from similar pulses do not yield optimal stimuli using calculus of variations is not surprising. There are many other final conditions (combinations of *V*, *m*, *h*, and *n*) that also lead to a spike. Thus we used the results for *V*, *m*, *h*, and *n* at the end of the depolarizing and hyperpolarizing pulses in Figure 2 as a starting point for additional simulations to determine waveforms that were optimal based on the RMS current metric. Specifically, we made small changes in one or more of the four parameters from their initial conditions for both the depolarizing and hyperpolarizing pulses and determined if these new values resulted in an *I_stim_*(*t*) waveform having a lower RMS current. This procedure was iterated repeatedly (a coordinate search) until we found local minima that we hypothesized do correspond to separate, optimal pathways for firing.

**Figure 5:**
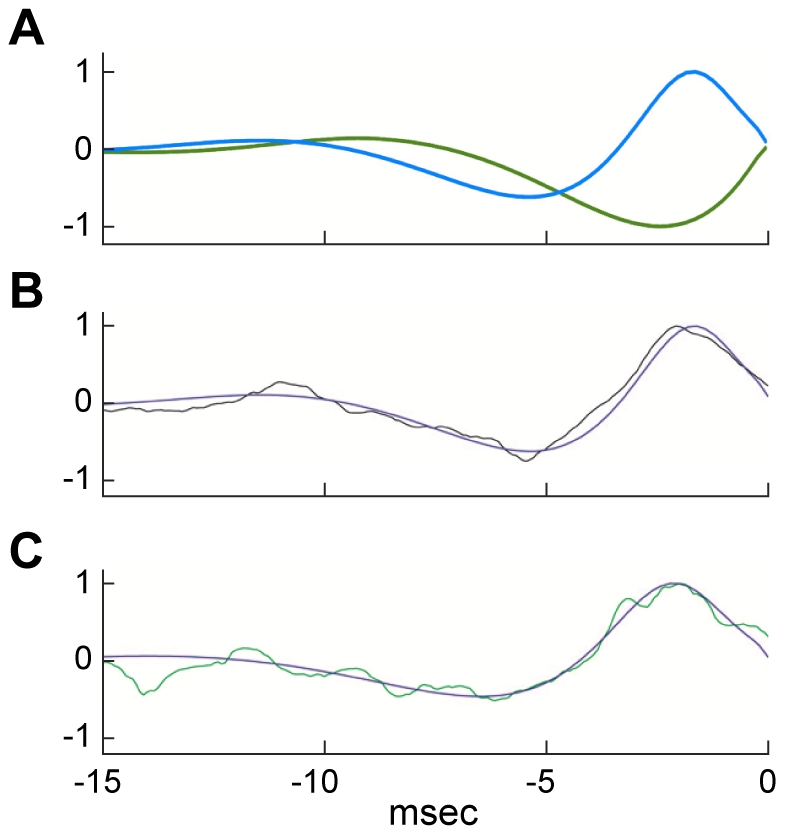
Comparison of optimal signals obtained from calculus of variation and noise analysis. **A**) Calculus of variation waveforms determined from an optimization of the *V*, *m*, *n*, and *h* values at the end of the depolarizing (blue curve) and hyperpolarizing (green curve) pulses used to elicit a spike in the Hodgkin & Huxley model (Figure 2B & C, respectively) as described in the text (Results and Methods). These results were normalized relative to the maximum value of the blue curve. The RMS current of the blue curve is 38% less than that of the green curve. **B**) Overlap of the blue curve in **A** with the optimal noise trace obtained from the analysis of Figure 3. **C**) Comparison of another one of the results from Figure 4 with the optimal calculus of variations stimulus determined from a revised version of the Hodgkin & Huxley equations [14].

The results of the analysis described above are illustrated in Figure 5A. The shapes of the new optimals depicted in blue and green are similar to their counterpoints in Figure 2B & C, respectively. The relative RMS currents of the curves in Figure 5A are different. Specifically, the RMS current of the blue curve is 38% less than that of the green curve, which suggests that it is the more optimal result. This waveform compares favorably with the optimal stimulus determined from the noise analysis in Figure 3, as shown in Figure 5B. Both of these results have a slight depolarizing phase in the early portion of each respective signal, a feature not apparent in all experimental results (Figure 4). Analysis comparable to that of Figure 5A on our modified version of the Hodgkin & Huxley model noted above [14] produced a waveform without the initial depolarizing phase (Figure 5C). The revised model provides an excellent description of some of our results (Figure 5C & Discussion).

We note that the theoretically derived waveforms in Figure 5B & C have not been tested experimentally to see if they optimally elicit spikes from the axon. We have determined waveforms experimentally that do elicit spikes, optimally, using the stochastic approach described in Figure 3. Those waveforms are very similar to our theoretical results as shown in Figure 5B & C, which suggests that the latter would also elicit spikes, optimally, from the experimental preparation. The similarity of results obtained from two very different approaches - one theoretical, the other experiments – provides a testable prediction of our theoretical work, a prediction that is well met based on the results in Figure 5B & C.

### Post-synaptic current polarity and optimality

**Figure 6:**
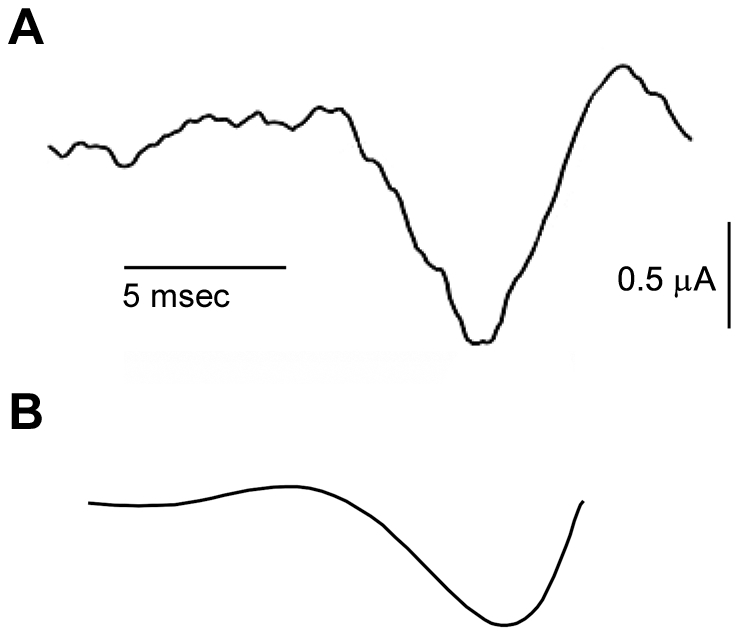
A) Optimal stimulus in the presence of purely inhibitory PSCs. This experiment was carried out as in Figure 3 except that the mixture of excitatory and inhibitory PSCs was replaced by an input consisting of only inhibitory PSCs. **B**) The optimized hyperpolarizing result from Figure 5A (green curve) is illustrated here showing similarity in stimulus shape compared to the noise based optimal derived from purely inhibitory PSCs.

The analysis of Figure 5A demonstrates two local minima of stimulus optimality. The more optimal of the two (blue curve) is consistent with the optimal stimulus obtained from the noise analysis in which both excitatory and inhibitory PSCs were used (Figure 5B & C). We hypothesized that the less optimal result (Figure 5A, green curve) might correspond to conditions in which only inhibitory PSCs were used. The results in Figure 6 describe an experimental test of this idea. The spike-triggered average current waveform for these conditions is illustrated in Figure 6A. We note that the depth of the hyperpolarizing phase is approximately twice as large as the depolarizing phase just prior to spike initiation. By contrast the amplitudes of these phases are approximately the same when mixed inhibitory and excitatory PSCs are used (Figure 4). These results provide evidence that the optimal stimulus for spike initiation depends upon overall state of inputs to the axon. We also note that the result is Figure 6A is qualitatively similar to the green curve in Figure 5A (also shown in Figure 6B) in that both have a slight depolarizing phase followed by a strong hyperpolarizing phase. Consequently experiments in which only inhibitory PSCs are used do appear to favor the less optimal of the two waveforms in Figure 5A.

### Post-synaptic current duration and optimality

**Figure 7:**
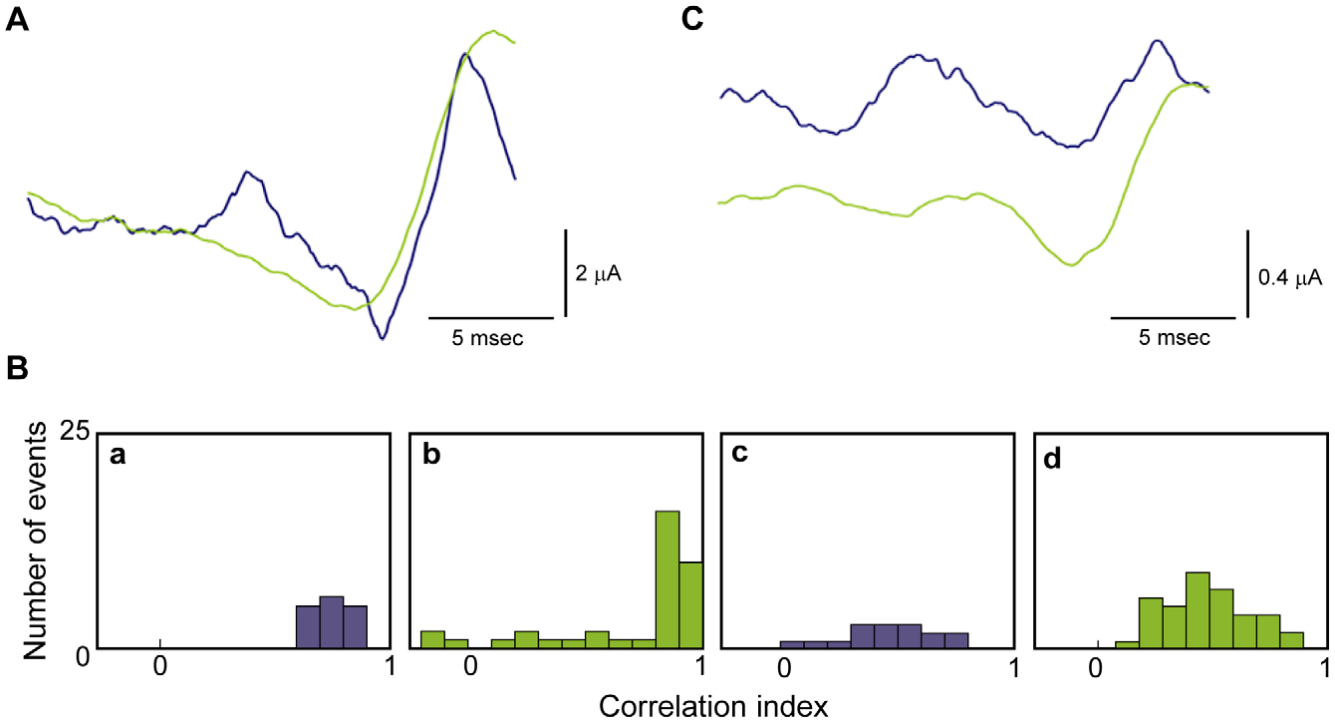
Effects of PSC duration on optimality. **A**) We repeated the experiments carried out in Figure 3 to determine the optimal shape for eliciting an action potential with PSCs having either a long (20 msec) decay constant (green curve) or short (1 msec) decay constant (blue curve). These signals are particularly different 5 to 10 msec prior to the action potential. During this region, short PSCs excite the neuron, whereas long PSCs inhibit the neuron. **B**) Correlation analysis of the short versus long PSC results as described in the text. **C**) The intracellular pH of the axon was elevated to increase axon excitability (15) and the experiments in **A** were repeated. For the axon with enhanced excitability, the difference between the optimal signals comprised of short PSCs and those comprised of long PSCs was more pronounced.

As noted above (Introduction) PSC duration can vary according to neuronal cell types ([12], and references therein). Since we have shown that the optimal input signal depends upon the type of inputs to the neuron (inhibitory vs excitatory) and that these optimal signals have different time scales (Figure 2), we hypothesized that short and long PSCs would optimally excite the neuron with different stimulus shapes. We repeated the experiments described in Figure 3 using a balanced combination of excitatory and inhibitory PSCs as in those experiments but with either a short (1 msec) or a long (20 msec) decay time constant. Figure 7A shows spike-triggered stimulus averages for the short (blue) and long (green) PSCs. Note that 5–10 milliseconds before a spike, the short PSC signal is excitatory (Figure 7A, blue), whereas the long PSC signal is inhibitory (Figure 7A, green). We tested the significance of the difference between the two results in Figure 7A using correlation analysis (Methods). The 20 msec portion of the noise signal preceding each spike in the short PSC experiment was correlated with the spike-triggered averaged signal from these experiments (blue trace in Figure 7A). These results shown in Figure 7B (panel **a**) are, not surprisingly, clustered close to a correlation value of one. A correlation of the 20 millisecond portion of the noise signal preceding each spike in the short PSC case with the spike-triggered average from the long PSC experiment (Figure 7B, panel **c**) gave correlation values between 0 and 1, indicating a poor correlation. A similar analysis of the 20 millisecond portion of the noise signal preceding each spike in the long PSC experiment correlated with the spike-triggered average determined from the long and short PSC cases are illustrated in Figure 7B, panels **b** and **d**, respectively.

To further explore the difference between short and long PSCs we increased the excitability of the axon, as demonstrated previously [15], by raising the internal pH and repeating the experiments described in Figure 3. We found that the optimal shape with short PSCs consisted of a growing sinusoidal stimulus with alternating periods of excitation and inhibition (Figure 7C). The long PSC signal consisted mainly of inhibition, with a less prominent superimposed sinusoidal fluctuation. Thus, the difference between short versus long PSCs appears to be more pronounced when the neuron has increased intrinsic excitability.

## Discussion

We have shown that a single neuron can be highly discriminatory for the shape of low amplitude stimuli that elicit an action potential and that the shape of the optimal stimulus is dependent upon input context, i.e., the optimal stimulus for eliciting a spike is determined by the nature and the type of all inputs to the neuron. Our results validate two methodologies to study optimality in neuronal systems. Using the calculus of variations, we determined optimal signals for the Hodgkin & Huxley model. This theory predicts that our stochastic search methodology derived from experiments should converge to the optimal stimulus derived from the theoretical approach, a prediction that is supported by the results in Figure 5B & C. Although optimality has been explored previously in simplified models [4], [11], [16], these are the first results using a complete ionic model of a neuron, which enabled us to demonstrate multiple mechanisms to elicit an action potential. Using a stochastic search methodology, we determined optimal signals in the squid giant axon. Unlike other studies that use spike-triggered averaging, we used minimally supra-threshold stimulation that is required to accurately determine optimal stimulus shapes [11], [17]. Careful titration of stimulus intensity to minimally suprathreshold levels enabled us to show that optimal shapes depend on the physiological context in which stimuli are presented. A novel feature of our analysis concerns two versions of the Hodgkin and Huxley model [13], [14]. The original version [13] predicts sustained firing of action potentials in response to a sustained, suprathreshold depolarizing current pulse. The axon preparation fires only once for these conditions, a result that is mimicked by our revised version of the model [14]. We applied the calculus of variations approach to both and found similar results (Figure 5B & C), which is not surprising since both models provide a good description of responses to brief duration pulses. The revised model provides a slightly improved description of our results compared to the original model (Figures 4 & 5) and the reduction in the oscillatory component of the theoretical results (Figure 5B & C) is consistent with the change from repetitive firing in the original version of the model in the response to long duration pulses (oscillatory behavior) compared to a single spike in the revised version for these conditions (absence of oscillations).

Our results indicate that questions of optimality are more complex than the one model-one optimal view that is widely found in the discussions of neuronal excitation. While simpler qualitative models which are more amenable to mathematical analysis than ionic models can also be used to qualitatively predict optimal signal, they may miss the multiple locally optimal signals that are needed to understand the full landscape of neuronal signaling. For example, an integrate-and-fire model does not predict post-inhibitory rebound excitation nor does it predict neuronal firing with inputs consisting solely of inhibitory PSCs. Multiple optimal signals could allow a neuron to be responsive to a wider range of stimuli, where stimulus context is key to understanding neuronal optimality. As further details of this context are considered [18], e.g. synaptic placement along a dendritic tree, both active and passive dendritic processing, synaptic facilitation/depression, all of which affect the temporal dynamics and polarity of the input stimulus to the soma, the role of separate firing mechanisms and multiple optimal signals will likely become even more important.

We have shown that PSC duration is an important factor in stimulus optimization (Figure 7). Further experiments could be carried out in which the duration of inhibitory PSCs are different than those of excitatory PSCs or the duration of either PSC type is itself a variable factor in the experiments. Additionally, we have relied on RMS current minimization as our criterion for stimulus optimization. Other minimization strategies could be used in future experiments such as one in which the rate of change of input current is used in conjunction with RMS current. Minimization of RMS current is directly relevant to deep brain stimulation protocols [7]–[9]. Its significance in other contexts such as sensory processing is less clear. For example, in the visual system quantum efficiency is perhaps the most relevant measure of optimality, i.e., the ability of an observer to detect a visual input with the fewest number of photons possible [5]. The relationship of RMS current input to this type of optimization is itself of topic of further research, as it the relationship of RMS current to stimulus optimization in other sensory modalities. Those studies, which have yet to be carried out, may demonstrate the relevance of the optimization of current shapes and current amplitude in the behavior of neural networks during information processing.

## Methods

### Theoretical

#### Hodgkin & Huxley model

The Hodgkin & Huxley model is given by
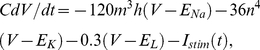
(1)where *C* is membrane capacitance (*C* = 1 µF/cm^2^), *V* is membrane potential in mV, *t* is time in msec, *E_Na_*, *E_K_*, and *E_L_* are the Nernst potentials for Na^+^, K^+^, and leak current, respectively, with *E_Na_* = 115 mV, *E_K_* = −12 mV, and *E_L_* = 10.613 mV, and *I_stim_* is the stimulus current in µA/cm^2^. The voltage- and time-dependent variables *m*, *n* and *h* in Equation (1) are dimensionless having values between 0 and 1. They are given by
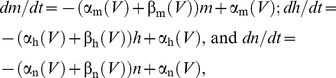
(2)with α_m_(*V*) = 0.1ϕ(25-*V*)/(exp(0.1(25-*V*))−1), β_m_(*V*) = 4 ϕexp(-*V*/18), α_h_(*V*) = 0.07 ϕexp(-*V*/20), β_h_(*V*) = ϕ/(exp(0.1(30- *V*))+1), α_n_(*V*) = 0.01ϕ(10-*V*)/(exp(0.1(10-*V*))−1), and β_n_(*V*) = 0.125 ϕexp(-*V*/80), where ϕ is a temperature parameter. All α's and β's are in msec^−1^. These equations were taken directly from Hodgkin & Huxley [13] with *V* replaced by -*V*, which is the modern sign convention for their model. This system of equations was used in the calculus of variations (following section), except for the results in Figure 5C in which β_n_ = 0.125 ϕexp(-*V*/80) was replaced with β_n_ = 0.125 ϕexp(-*V*/20), as described in previous work from this laboratory [14]. The membrane potential *V* in Figure 2 and elsewhere in this report was replaced by *V*+60, which is also consistent with modern usage of the Hodgkin & Huxley model. They assumed rest potential was 0 mV, whereas −60 mV is found in most neurons. The temperature parameter ϕ was set to 1.5 to match the temperature of our experiments.

#### Calculus of variations

We minimize the L^2^ norm of the applied current *I_stim_*(*t*) to the Hodgkin & Huxley model. Following earlier work [11], this procedure yields the following function to minimize:
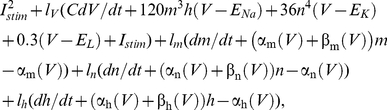
(3)where *l_V_*, *l_m_*, *l_n_*, and *l_h_* are Lagrange multipliers. The Euler equations [10], [11] were applied yielding the following
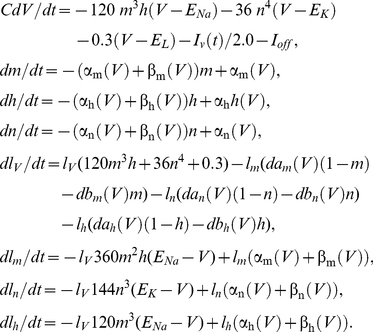
(4)The offset current *I_off_* was chosen as 0 except where otherwise indicated. The Hodgkin & Huxley model was started at rest. The model was stimulated with 4 msec duration depolarizing current pulses having increasing amplitude until an action potential was initiated (Figure 2). The initial (resting) values of the Hodgkin & Huxley parameters were *V* = .0036 mV, *m* = 0.0530, *n* = 0.3177, *h* = 0.5960, *l_V_* = 0.0001676, *l_m_* = 0.001386, *l_n_* = 0.2044, and *l_h_* = 0.09389 (state α_0_ in Figure 2). When calculating optimal stimuli corresponding to post-inhibitory rebound, the last four parameters were changed to *I_V_* = −0.0188, *l_m_* = 0.3138, *I_n_* = 15.4266, and *I_h_* = 11.3144. Slightly different parameters were used for Figure 5Csince this simulation used our revised Hodgkin& Huxley model. For final values of the parameters (state β_1_ in Figure 2) we used results corresponding to the end of a slightly suprathreshold 4 msec depolarizing pulse, i.e., *V* = 7.91 mV, *m* = 0.1173, *n* = 0.3548, *h* = 0.5954. We used Matlab's bvp4c function (MathWorks; Natick, MA) to find the optimal stimulus over 20 msec in duration (running the optimization for 40 msec prior to AP did not change the results) that brought the neuron from rest, α_0_, to β_1_. Similar results were found with Mathematica (Wolfram Research; Champaign, Il) using a shooting method. For post-inhibitory rebound (PIR) stimuli, we repeated the above methods with a hyperpolarizing pulse 10 msec in duration. As noted above (Results), this procedure gave signals (Figure 2B & C) that did not closely match the optimal stimulus obtained from noise analysis (Figure 3C). We subsequently used the *V*, *m*, *n*, and *h* sets corresponding to the end of the pulses in Figure 2 as starting points for refinement of the results. A small change was made in any one of the parameters and calculus of variations simulations were performed. The RMS current of the result was used as the determining factor in a coordinate search in 4 parameter space (*V*, *m*, *n*, *h*) for a local optimal of action potential initiation. These local minima were verified by slightly perturbing the final state and checking that the search method returned to each respective local minima. The final results of this analysis are illustrated in Figure 5A.

#### Convolutions and correlations

Let d be the input data and s be the proposed optimal stimulus scaled so that 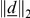 = 1. We consider two important quantities. Our measure of how similar d is to s is d•s/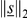 was used in Figure 7. This quantity has the geometric interpretation of the cosine of the angle between d and s. Here 0 means that the signals are orthogonal to each other and −1 or 1 indicates that the signals are the same except for a possible scaling constant. We also used s to predict whether an action potential would occur after a neuron had been presented with signal d. This is done with the classical LNP nonlinear Poisson model that proposes that the rate of firing an action potential is a function of d•s. We used d•s to predict the firing rate of an action potential in Figure 3.

### Experimental

Experiments were carried out on squid giant axons using methods previously described [15]. Stochastically varying current was administered to the axon for 10 sec periods using stimulus profiles generated by computer (MatLab) of a simple model of stochastically summated polysynaptic currents (PSCs). Excitatory and inhibitory PSCs were generated independently, each with a Poisson rate having a mean of 10 events per msec. Each PSC had an exponential rise time constant of 0.25 msec and decay time constant of 1 msec [15]. These parameters were used in all runs unless otherwise noted. The stimulus profile was the sum at any moment of all PSCs. The overall intensity of the stimulus was varied by changing the amplitude of all PSCs. The computed stimulus profiles were converted to an analog stimulus using a D-A converter (National Instruments, Austin, TX) controlled by software (LabView 6, National Instruments). The mean current for any run was zero because the excitatory and inhibitory PSCs had identical profiles and Poisson distributions. The exception was the experiment described in Figure 6 for which only inhibitory PSCs were used.

## Supporting Information

Figure S1 **A**) Optimal *I_stim_*(*t*)for eliciting a spike from the Hodgkin & Huxley model corresponding to a depolarizing current pulse as in Figure 2 in the text – exogenous stimulation. **B**) Optimal waveform for eliciting a spike as In **A**, but with *I_stim_*(*t*) determined by Equations S1–S3 with *E_syn_* = 25 mV. **C**) Curves in **A** and **B** shown superimposed.
(TIF)

Figure S2 **A**–**B**) Similar analysis as in Figure S1 with *E_syn_* = −25 mV. The curves in **A** and **B** are shown superimposed in **C**.
(TIF)

Text S1 Application of the calculus of variations to endogenous stimulation.
(DOC)

## References

[1] Mainen ZF, Sejnowski TJ (1995). Reliability of spike timing in neocortical neurons.. Science.

[2] Koch C (1999). Biophysics of Computation: Information Processing in Single Neurons.

[3] Niven JE, Laughlin SB (2008). Energy limitation as a selective pressure on the evolution of sensory systems.. J Exp Biol.

[4] Jezernik S, Morari M (2005). Energy-optimal electrical excitation of nerve fibers.. IEEE Tran on Biomed Eng.

[5] Watson AB, Barlow HB, Robson JG (1983). What does the eye see best?. Nature.

[6] Brenner N, Bialek W, de Ruyter van Steveninck R (2000). Adaptive rescaling maximizes information transmission.. Neuron.

[7] Feng XJ, Greenwald B, Rabitz H, Shea-Brown E, Kosut R (2007). Toward closed-loop optimization of deep brain stimulation for Parkinson's disease: concepts and lessons from a computational model.. J Neural Eng.

[8] Feng XJ, Shea-Brown E, Greenwald B, Kosut R, Rabitz H (2007). Optimal deep brain stimulation of the subthalamic nucleus–a computational study.. J Comp Neurosci.

[9] Rubin JE, Terman D (2004). High frequency stimulation of the subthalamic nucleus eliminates pathological thalamic rhythmicity in a computational model.. J Comp Neurosci.

[10] Gelfand IM, Fomin SV (1963). Calculus of Variations.

[11] Forger DB, Paydarfar D (2004). Starting, stopping, and resetting biological oscillators: in search of optimum perturbations.. J Theor Biol.

[12] Reyes A (2001). Influence of dendritic conductances on the input-output properties of neurons.. Ann Rev Neurosci.

[13] Hodgkin AL, Huxley AF (1952). A quantitative description of membrane current and its application to conduction and excitation in nerve.. J Physiol.

[14] Clay JR, Paydarfar D, Forger DB (2008). A simple modification of the Hodgkin and Huxley equations explains type 3 excitability in squid giant axons.. J R Soc Interface.

[15] Paydarfar D, Forger DB, Clay JR (2006). Noisy inputs and the induction of on-off switching behavior in a neuronal pacemaker.. J Neurophysiol.

[16] Moehlis J, Shea-Brown E, Rabitz H (2006). Optimal inputs for phase models of spiking neurons.. J Comp Nonlinear Dynamics.

[17] Pillow JW, Simoncelli EP (2006). Dimensionality reduction in neural models: An information-theoretic generalization of spike-triggered average and covariance analysis.. J Vision.

[18] Abbott LF, Regehr WG (2004). Synaptic computation.. Nature.

