# Supplementary figures and images for: Optimal Stimulus Shapes for Neuronal Excitation
Source: PLoS Comput Biol. 2011 Jul 7;7(7):e1002089. doi: 10.1371/journal.pcbi.1002089 (PMC3131391; doi:10.1371/journal.pcbi.1002089)

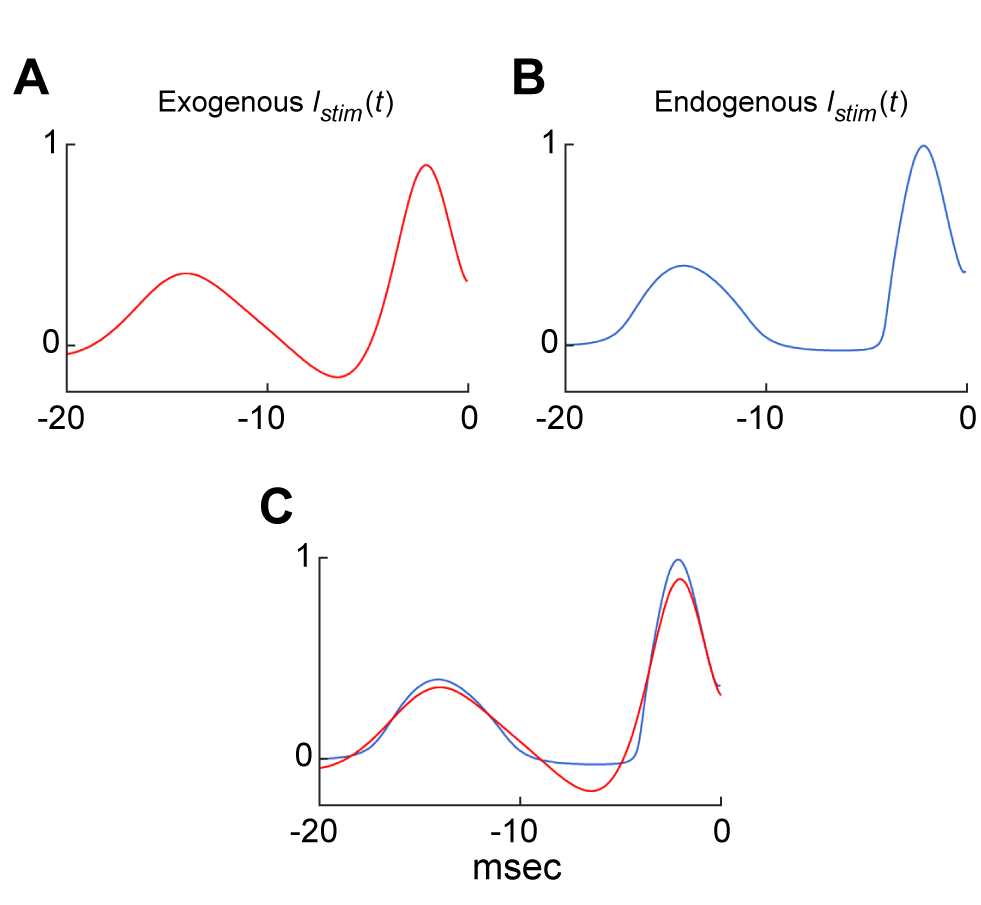

Supplement: Figure S1 — A) Optimal Istim(t)for eliciting a spike from the Hodgkin & Huxley model corresponding to a depolarizing current pulse as in Figure 2 in the text – exogenous stimulation. B) Optimal waveform for eliciting a spike as In A, but with Istim(t) determined by Equations S1–S3 with Esyn = 25 mV. C) Curves in A and B shown superimposed. (TIF) [file pcbi.1002089.s001.tif]

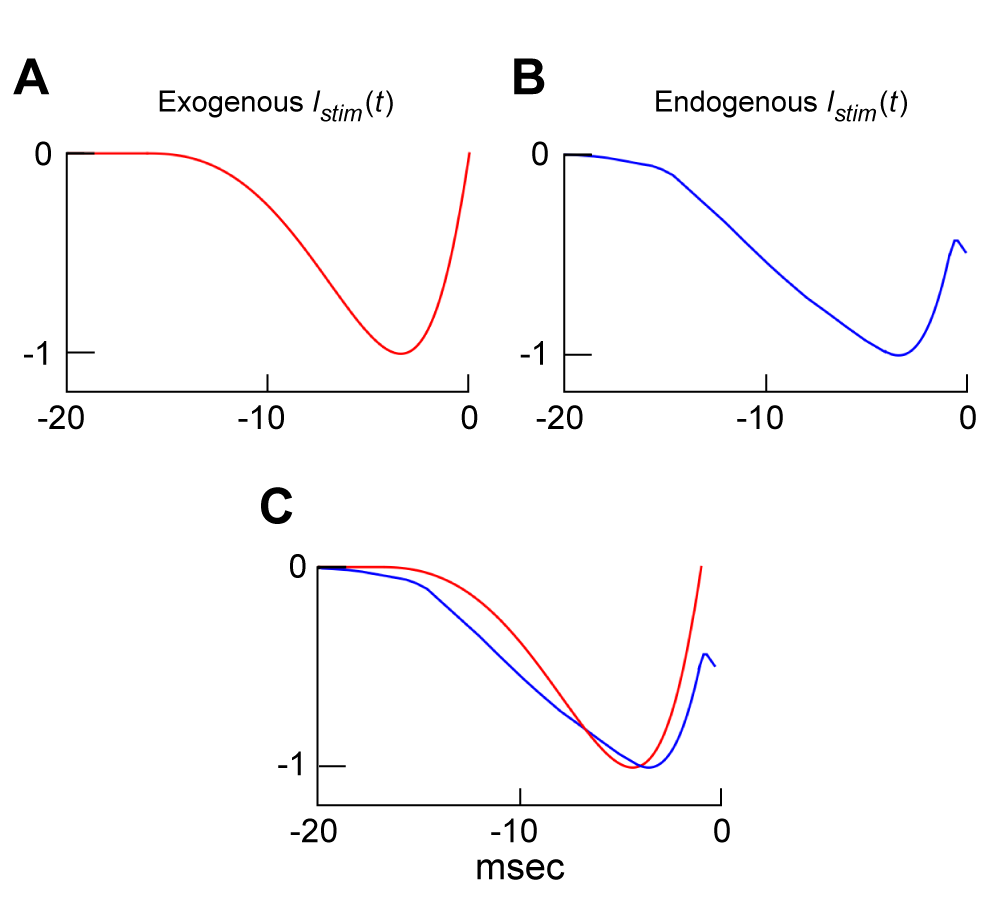

Supplement: Figure S2 — A–B) Similar analysis as in Figure S1 with Esyn = −25 mV. The curves in A and B are shown superimposed in C. (TIF) [file pcbi.1002089.s002.tif]
