# Supplementary material for: Optimal Stimulus Shapes for Neuronal Excitation
Source: PLoS Comput Biol. 2011 Jul 7;7(7):e1002089. doi: 10.1371/journal.pcbi.1002089 (PMC3131391; doi:10.1371/journal.pcbi.1002089)
Supplement: Text S1 — Application of the calculus of variations to endogenous stimulation. (DOC) [file pcbi.1002089.s003.doc]

Text S1. Supplement to “Optimal stimulus shapes for neuronal excitation”

Daniel B. Forger^1,5^, David Paydarfar^2,3,5^, John R. Clay^4,5^

^1^Department of Mathematics and Center for Computational Medicine and Bioinformatics,

University of Michigan, Ann Arbor, MI

^2^Department of Neurology and Physiology, University of Massachusetts Medical School, Worcester, MA

^3^Wyss Institutes for Biologically Inspired Engineering, Harvard University, Boston, MA

^4^National Institute of Neurological Disorders and Stroke, National Institutes of Health, Bethesda, MD

^5^Marine Biological Laboratory, Woods Hole, MA

**Calculus of variations for endogenously applied currents**

A neuron in the brain is embedded within a network of neurons that are interconnected synaptically. In this section we determine optimal waveforms for stimulating a neuron by requiring that *I_stim_*(*t*) be given by *g_syn_*(*t*) (*V*(*t*) - *E_syn_*), where *g_syn_*(*t*) is a particular type of synaptic conductance, either excitatory or inhibitory, and *E_syn_* is the reversal potential of the conductance [1]. As noted in the text, *I_stim_*(*t*) can have both positive and negative phases when it is unconstrained to provide the optimal pathway to spike threshold - exogenous stimulation (Results in text). When *I_stim_*(*t*) is constrained to be given by PSCs, one requirement of the analysis is that *g_syn_*(*t*) cannot be less than 0. We restricted *g_syn_*(*t*) to nonnegative values by using the factor exp(*g_syn_*(*t*) z)/(1+exp(*g_syn_*(*t*) z)), where z is a constant having units of kΩ⋅cm^2^.

(Conductance in the Hodgkin & Huxley model has units of mS/cm^2^). This factor is near zero when *g_syn_*(*t*) is below zero and is sufficiently smooth as a function of time to allow it to be numerically tractable [2]. Any potentially negative portion of the signal is minimized by choosing z to be relatively large. We used z = 50 kΩ⋅cm^2^. Applying the Euler equations yields

2*g_syn_*(*t*) = *dI_stim_*(*t*)/*dg_syn_*(*t*), (S1)

*g_syn_*(*t*)= (*I_V_*(*E_syn_* - *V*(*t*))) exp(z *g_syn_*(*t*))(1 + exp(z *g_syn_*(*t*)) + z *g_syn_*(*t*))/(1 + exp(z *g_syn_*(*t*)))^2^. (S2)

The above equations were solved by Newton’s method with an initial guess of (*I_V_*(*E_syn_* - *V*(*t*))/2) where we required a relative error of less than e^-6^. The rest of the Euler equations are a before (Methods in text)

except for the following

*dI_V_*/*dt* = *I_V_*(120 *m*^3^*h* + 0.3 + exp(*g_syn_*(*t*)z)/(1+exp(*g_syn_*(*t*)z)) - *I_m_*(*d*α_m_(*V*)(1-*m*) -

*d*β_m_(*V*)*m*) - *I_n_*(*d*α_n_(*V*)(1-*n*) - *d*β_n_(*V*)*n*) – *I_h_*(*d*α_h_(*V*)(1-*h*) - *d*β_h_(*V*)*h*). (S3)

Calculations were carried out to determine optimal signals to bring the model to *V* = 7.2 mV, *m* = 0.112, *n* = 0.3485, and *h* = 0.5435 for *E_syn_* = 25 mV, and *V* = -3 mV, *m* = 0.035, *n* = 0.270, and *h* = 0.68 for *E_syn_* = -25 mV. The results are shown in Figures S1 and S2. For each condition the results are compared with the corresponding *I_stim_*(*t*) obtained for exogenous stimulation as described in Figure 2 of the main text. These two contrasting approaches yield waveforms for the optimal stimulus for eliciting a spike that do not differ substantially, especially for excitatory PSCs (Figure S1).

**REFERENCES**

1. Destexhe A, Mainen ZF, Sejnowski TJ (1994) Synthesis of models for excitable membranes, synaptic transmission and neuromodulation using a common kinetic formalism. J Comp Neurosci 1:195-230.
2. Forger DB, Paydarfar D (2004) Starting, stopping, and resetting biological oscillators: In search of optimum perturbations. J Theor Biol 230:521-532.
